# Identification of self-incompatibility in macadamia *(Macadamia* SPP.*)* using field-bagging and fluorescence microscopy

**DOI:** 10.3389/fpls.2026.1771293

**Published:** 2026-04-16

**Authors:** Guanghong Kong, Wencai Yu

**Affiliations:** Macadamia Agricultural Engineering Research Center of Yunnan Province, Yunnan Institute of Tropical Crops, Jinghong, China

**Keywords:** autogamy, field-bagging technology, fluorescence-microscopy technology, macadamia, self-fertilization, self-incompatibility, self-pollination

## Abstract

Self-incompatibility (SI) significantly reduces crop yield, often far below its genetic potential. Developing self-compatible varieties is the most effective strategy for overcoming SI in crops. Most macadamia (*Macadamia* SPP.) species exhibit SI or partial self-incompatibility (PSI), so the efficient identification of self-compatible germplasms has emerged as a crucial topic. To characterize self-incompatibility phenotypes in macadamia germplasm resources, we conducted a four-year field study using the field-bagging method and established a standardized classification system of self-incompatibility. That is, the degree of SI was based on the final self-incompatibility index (F_SI), which was calculated based on the open-pollination final nut set per raceme (OP_FNS) and self-pollination final nut set per raceme (SP_FNS) values (strong SI: F_SI ≥ 0.7, medium SI: 0.4 ≤ F_SI <0.7, and weak SI: F_SI < 0.4). To develop a method for identifying macadamia SI more efficient and cost-effective than the field-bagging method, we employed pollen tube fluorescence staining, where the degree of SI was classified based on the percentage of pistils for which the pollen tube length exceeded 7/10 of the style (PLS) (strong SI: PLS ≤ 16%, medium SI: 16% < PLS ≤ 35%, and weak SI: PLS > 35%). Through comprehensive analysis of the field-bagging and fluorescence-microscopy observations, thirteen varieties with strong SI (816, 778, 842, Special, 812, D, 820, 246, 772, A16, A4, 951, and 695), six varieties with moderate SI (851, 828, 508, 936, O.C, and D4), and four varieties with weak SI (915, HY, 836, and 814) were identified. Our study provides a theoretical foundation and technical support for advancing germplasm resource innovation, and the genetic improvement and breeding of self-compatible macadamia varieties.

## 1 Introduction

Self-incompatibility (SI) significantly reduces crop yield, and almost all self-incompatible crop species exhibit pollen limitation issues (Burd, 1994; Larson and Barrett, 2000; Garibaldi et al., 2011; Trueman et al., 2022). Self-incompatible, economically important crops require pollination trees and pollinators (insects or artificial) during agricultural production (Potts et al., 2016), and cross-pollination can only be achieved when compatible pollen is successfully transferred to the stigma by pollinators (Potts et al., 2016; Grass et al., 2018; Trueman et al., 2022). Despite the presence of pollination trees and an adequate number of beehives, self-incompatible crops may still face the problem of insufficient pollination (Grass et al., 2018; Trueman et al., 2022). At present, both the number and diversity of wild pollinators are experiencing a sharp decline, exacerbating this problem in self-incompatible crop species (Aizen and Harder, 2009; Gallai et al., 2009; Potts et al., 2010; Garibaldi et al., 2011; Potts et al., 2016; LeBuhn and Vargas Luna, 2021). Although supplementary artificial pollination can increase the average crop yield by 63% (Knight et al., 2005; Bennett et al., 2020), the challenges of labor shortages and high costs remain difficult to address. In the long term, crop SI may become a major constraint for the sustainable development of related industries.

Self-incompatible plants that have long experienced pollen limitation may evolve self-compatibility as a strategy to ensure successful reproduction (Pannell and Barrett, 1998; Busch, 2011). Researchers have found that such evolutionary transitions have occurred multiple times across different plant lineages (Steinbachs and Holsinger, 2002; Igic et al., 2004). To avoid pollen limitation, many plants have evolved partial self-incompatibility (PSI), which may serve as an adaptive mechanism to overcome reproductive barriers (Vallejo-Marín and Uyenoyama, 2004; Zhao et al., 2022). Numerous studies have demonstrated that, within a given self-incompatible crop species, most varieties exhibit SI, while a small subset exhibit self-compatible variants, for example, sweet cherry (Ushijima et al., 2004; Sonneveld et al., 2005; Marchese et al., 2007; Cachi and Wünsch, 2011), apricot (Vilanova et al., 2006; Wu et al., 2010), almond (Matsumoto and Tao, 2019), pear (Qi et al., 2011), citrus (Liang et al., 2020), and pummelo (Hu et al., 2022). The selection pressure from changes in the external environment has led to frequent losses and reacquisitions of SI during natural evolution across angiosperms (Zhao et al., 2022). Therefore, identifying self-compatible mutant materials and systematically evaluating their compatibility both contribute to addressing the issue of insufficient pollination in agricultural production, and provide valuable insights for breeding and genetic improvement efforts.

Macadamia demonstrates either complete SI or partial self-incompatibility (PSI), and pollen limitation is extremely severe, which results in low yields (Wallace et al., 1996; Knight et al., 2005; Trueman et al., 2022). A large number of studies have shown that the yield and quality of macadamia are thus heavily dependent on cross-pollination (Sedgley et al., 1985; Howlett et al., 2019; Kaur et al., 2024; Trueman et al., 2024). Overcoming crop SI is an effective strategy for achieving high yield and quality, and breeding self-compatible varieties represents the most effective method for this purpose (Tsukamoto et al., 1999; Ahmad et al., 2022). Therefore, the identification and evaluation of naturally occurring self-compatible variation resources are of crucial importance for breeding self-compatible varieties (Mable, 2008; Li et al., 2023). Previous studies have demonstrated that different varieties of macadamia exhibit variation in SI phenotypes, with distinct levels of SI. To investigate the SI characteristics of different macadamia varieties, field-bagging experiments have been performed by many researchers (Howlett et al., 2015; Sedgley et al., 2015; Howlett et al., 2019; Langdon et al., 2019; Richards et al., 2020; Kaur et al., 2024). The results clearly demonstrated distinct SI patterns among macadamia varieties. Specifically, varieties 741, 791, and 508 showed weak SI (which indicates strong self-compatibility), whereas varieties 344, A4, A16, Daddow, and 816 displayed strong SI (suggesting weak self-compatibility) (Sedgley et al., 2015; Langdon et al., 2019; Richards et al., 2020; Kaur et al., 2024).

To identify SI in macadamia, the field-bagging method is typically used, in which cross-pollination is isolated by bagging and the degree of SI is assessed based on the final nut set rate. The higher the nut set rate, the weaker the SI; conversely, the lower the nut set rate, the stronger the SI (Howlett et al., 2015, Howlett et al., 2019; Richards et al., 2020; Kaur et al., 2024). The final nut set rate can be influenced by various factors such as environmental conditions, tree nutritional status, and pest and disease pressure (Stephenson and Cull, 1986; Stephenson et al., 1989; Olesen et al., 2008; Trueman, 2013; Herbert et al., 2019; Howlett et al., 2019); thus, the nut set rate of the same variety varies significantly across different years, leading to significant annual fluctuations in SI. Additionally, the SI of macadamia cannot be reliably determined based on a single nut set rate observed within a single year. As such, the method of identifying the SI status of different varieties of macadamia through field-bagging isolation using the cross-flower pollen method has a large workload, high cost, long cycle, and is easily affected by internal and external factors. We systematically characterized the self-incompatibility of macadamia using field-bagging over several years, and established a quantitative standard for evaluating the degree of self-incompatibility. We also developed an economical, rapid, and accurate method for identifying macadamia SI --fluorescence-microscopy. Our results will provide a theoretical basis and technical support for the evaluation of germplasm resources and the breeding of new self-compatible varieties of macadamia.

## 2 Materials and methods

### 2.1 Materials

A macadamia variety comparison test field was arranged in 2003 at the experimental field of Yunnan Institute of Tropical Crops (Jinghong, Yunnan, China), and a total of 36 macadamia varieties were planted in the orchard using a randomized complete block design with three blocks, six trees per variety per replicate. In this study, 33 varieties 22-year-old macadamia plants were selected as test materials with consistent management practices, similar growth conditions, and no signs of pests or diseases. Detailed information on the varieties is presented in **Table 1**.

**Table 1:** Information for the macadamia varieties used in this study.

Order number	Variety name	Abbreviation	Type of germplasm	Variety origin
1	HY	HY	*M. integrifolia*/*M. teraphylla* Hybrids	Australia
2	HVA16	A16	*M. integrifolia*/*M. teraphylla* Hybrids	Australia
3	HVA4	A4	*M. integrifolia*/*M. teraphylla* Hybrids	Australia
4	Beaumont/HAES695	695	*M. integrifolia*/*M. teraphylla* Hybrids	Australia
5	Special	Special	*M. integrifolia*	Australia
6	Own Choice	O.C	*M. integrifolia*	Australia
7	Renown	D4	*M. integrifolia*/*M. teraphylla* Hybrids	Australia
8	D	D	*M. integrifolia*	Australia
9	HAES842	842	*M. integrifolia*	America
10	HAES904	904	*M. integrifolia*	America
11	HAES851	851	*M. integrifolia*	America
12	HAES918	918	*M. integrifolia*	America
13	HAES749	749	*M. integrifolia*	America
14	HAES828	828	*M. integrifolia*	America
15	HAES836	836	*M. integrifolia*	America
16	HAES816	816	*M. integrifolia*	America
17	HAES932	932	*M. integrifolia*	America
18	HAES936	936	*M. integrifolia*	America
19	HAES906	906	*M. integrifolia*	America
20	HAES762	762	*M. integrifolia*	America
21	HAES863	863	*M. integrifolia*	America
22	HAES820	820	*M. integrifolia*	America
23	HAES778	778	*M. integrifolia*	America
24	HAES951	951	*M. integrifolia*	America
25	HAES849	849	*M. integrifolia*	America
26	HAES915	915	*M. integrifolia*	America
27	HAES508	508	*M. integrifolia*	America
28	Keauhou/HAES246	246	*M. integrifolia*	America
29	HAES772	772	*M. integrifolia*	America
30	HAES948	948	*M. integrifolia*	America
31	HAES861	861	*M. integrifolia*	America
32	HAES814	814	*M. integrifolia*	America
33	HAES812	812	*M. integrifolia*	America

### 2.2 Overview of the experimental site

All the test materials were obtained from the comparative test base for macadamia varieties at the Yunnan Institute of Tropical Crops, located in Jinghong, Yunnan Province, China (100°30’E, 21°50’N). The site has a perennial frost-free climate, with an annual average rainfall of 948–1515 mm, primarily concentrated between May and October, at an altitude of 560–600 m. The soil is red soil with a deep soil layer and a pH range of 4.5–5.5.

### 2.3 Identification of self-incompatibility in macadamia using the field-bagging method

#### 2.3.1 Experimental design

The assessment of self-incompatibility in macadamia using field-bagging method is affected by the biennial bearing. In addition, this approach also is influenced by several confounding factors, including variations in flowering phenology, biotic and abiotic stresses, and weather conditions during anthesis. Therefore, reliable determination of self-incompatibility levels via field-bagging necessitates comprehensive analysis based on multi-year experimental data. In this study, we evaluated the self-incompatibility of 33 macadamia varieties from 2021 to 2024. The number of varieties assessed each year was as follows: eleven in 2021, twenty-six in 2022, fifteen in 2023, and twenty-three in 2024 (**Supplementary Table 1**). Finally, the self-incompatibility levels of these varieties were determined based on evaluations conducted over at least two years and the confirmation of consistent self-incompatibility intensities across those years.

Self-pollination can be divided into two types: autogamy, where pollination occurs within the same flower, or geitonogamy, in which pollination occurs between different flowers of the same plant (Eckert, 2000; Kaur et al., 2024). Geitonogamy can occur due to pollination between flowers of the same raceme, between different racemes of the same plant, or between different racemes of the same genotype (Eckert, 2000). In this study, only one self-pollination method—autogamy—was employed. However, the pollen source included both the same floret and other florets within the same raceme.

**Figure 1:**
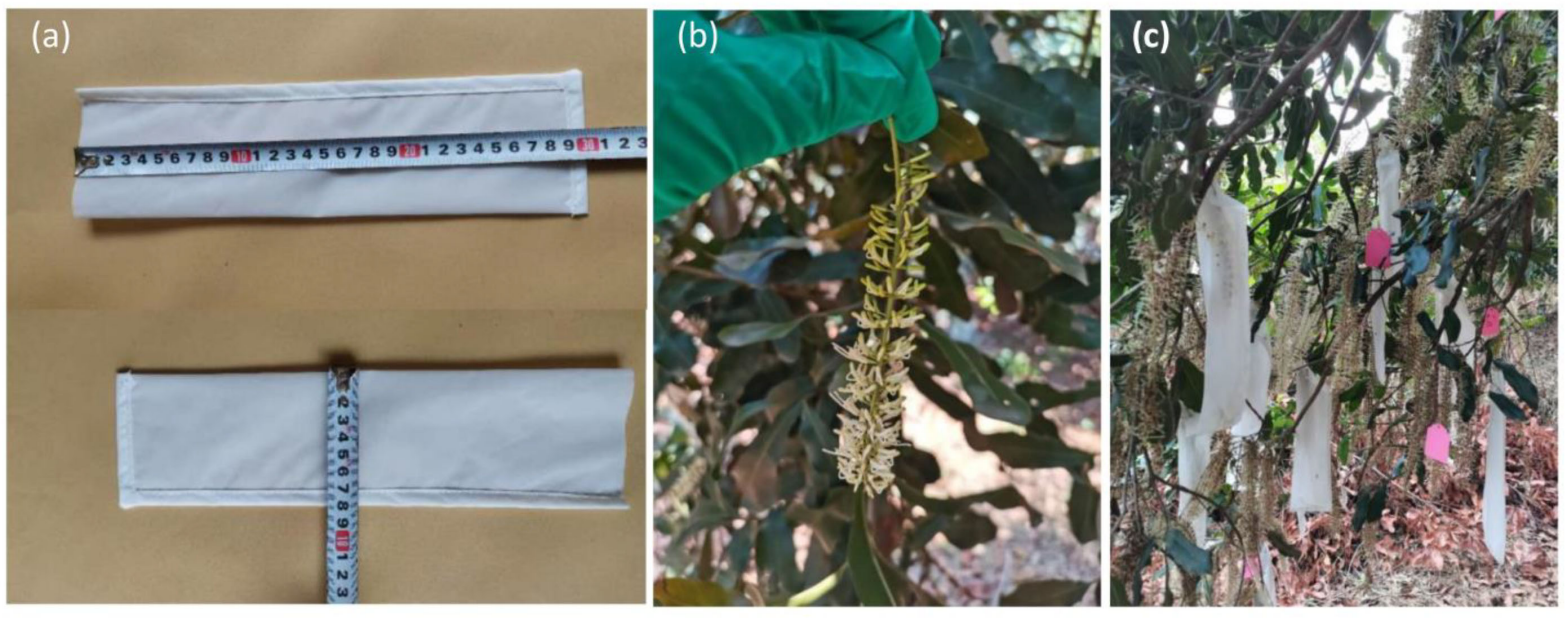
Isolation pollination of macadamia using the field-bagging method. **(a)** The specified nylon mesh bag; **(b)** macadamia racemes; and **(c)** isolation pollination via bagging.

A nylon mesh bag with a pore size of 500 mesh (25 μm) and dimensions of 30×7 cm was used for self-pollination. The nylon mesh bag was breathable and light- and water-permeable, allowing it to isolate pollinators and foreign pollens falling into the stigma without affecting fruit growth and development (**Figure 1a**). Before bagging, uncracked florets (young flowers) and opened florets (old flowers) were manually removed to ensure that remaining florets exhibited cracked sepals that were not yet rolled back (**Figure 1b**). After bagging, the bag opening was tightened, fixed with a stapler to ensure that it did not fall off, and each bag was labeled accordingly (**Figure 1c**). After 20 days, the mesh bags were removed and the initial nut setting number was recorded. The final nut setting number was counted 150 days later. The open-pollination raceme was used as the control; the number of test racemes is presented in **Supplementary Table 1**.

#### 2.3.2 Parameters and calculation formulas for self-incompatibility identification in macadamia

The initial nut set per raceme (INS), final nut set per raceme (FNS), abscission rate (AR), initial self-incompatibility index (I_SI), final self-incompatibility index (F_SI), open-pollination initial nut set per raceme (OP_INS), self-pollination initial nut set per raceme (SP_INS), open-pollination final nut set per raceme (OP_FNS), and self-pollination final nut set per raceme (SP_FNS) were used to evaluate the self-incompatibility of macadamia. Open-pollination was used as the control. The calculation formulas are shown as follows:


$$\text{INS}=\frac{\text{ initial nut set}}{\text{number of tested racemes}}$$

$$\text{FNS}=\frac{\text{final nut set }}{\text{number of tested racemes}}$$

$$\text{AR}=\frac{\text{INS}-\text{FNS}}{\text{INS}}\times 100\%$$

$$\text{I}\_\text{SI}=\frac{\text{OP}\_\text{INS}-\text{SP}\_\text{INS}}{\text{OP}\_\text{INS }}$$

$$\text{F}\_\text{SI}=\frac{\text{OP}\_\text{FNS}-\text{SP}\_\text{FNS}}{\text{OP}\_\text{FNS}}$$

### 2.4 Identification of self-incompatibility in macadamia using the fluorescence-microscopy method

#### 2.4.1 Sample preparation and fluorescence-microscopy observations

Prior to flowering, racemes of various macadamia varieties were enclosed in nylon bags, with the treatment and bagging protocols following the same procedures as those employed in the field-based bagging isolation pollination method. Nine days later, the nylon mesh bags were removed, and the raceme pedicels were gently tapped with a pencil to detach pistils that were not firmly attached to the raceme axis. All retained pistils were then collected and prepared for microscopic analysis. First, the pistils were immersed in an 89:6:5 FAA solution (89% ethanol, 6% glacial acetic acid, and 5% formaldehyde) for 24 h (Kong et al., 2023a). Subsequently, they were washed consecutively in 60%, 40%, and 20% ethanol solutions for 3 minutes each, with a final wash of 8–10 minutes in ultrapure water. Next, the pistils were softened by soaking in 2 M NaOH at 28–30 °C for 2.5 h. The softened pistils were washed twice for 5–8 min in ultrapure water, then transferred into phosphate buffer (pH, 7.0). The softened pistils were longitudinally sectioned using a surgical blade and placed on microscope slides for staining. Staining was performed using a 0.1% aniline blue staining solution (pH 10) for 30 s. Finally, pollen tube growth was observed under ultraviolet (UV) spectrum using a fluorescence microscope (DM6 B, Leica, Wetzlar, Germany), and images were captured through channel A using a 100× UV light filter.

#### 2.4.2 Growth characteristics, evaluation parameters, and calculation formulas for the macadamia pollen tubes

After pollination, pollens germinate and the pollen tubes grow through the outer cell layers and into the stigma over approximately one to two days. The pollen tubes continue to grow though the style, needing seven to nine days to reach the ovary (Sedgley, 1983; Sedgley et al., 1985; Kong et al., 2023a). We classified the growth behavior of pollen tubes into four types. Type I: pollen tubes were not observed in either the stigma or the style (NPT). Type II: the pollen tube length (PTL) was less than two-tenths of the total pistil length (PTL ≤2/10, US), which means that the pollen tubes stopped growing in the upper part of the style. Type III: the pollen tube length ranged from two-tenths to seven-tenths of the total pistil length (2/10 < PTL ≤ 7/10, MS), indicating that the pollen tubes grew to the middle of the style. Type IV: the pollen tube length was greater than seven-tenths of the total pistil length (PTL > 7/10, LS); that is, the pollen tubes grew to the lower half of the style or entered the ovary. We introduced several parameters to evaluate self-incompatibility in macadamia germplasms: the number of pistils utilized for observation (NPO), the number of ovaries with a single ovule (SO), the number of ovaries with double ovules (DO); the percentage of NPT (PN), the percentage of US (PUS), the percentage of MS (PMS), the percentage of LS (PLS), the percentage of ovaries with a single ovule (PSO), and the percentage of ovaries with double ovules (PDO). The corresponding calculation formulas are as follows:


$$\text{PN}=\frac{\text{NPT}}{\text{NPO}}\times 100\%$$

$$\text{PUS}=\frac{\text{US}}{\text{NPO}}\times 100\%$$

$$\text{PMS}=\frac{\text{MS}}{\text{NPO}}\times 100\%$$

$$\text{PLS}=\frac{\text{LS}}{\text{NPO}}\times 100\%$$

$$\text{PDO}=\frac{\text{DO}}{\text{NPO}}\times 100\%$$

$$\text{PSO}=\frac{\text{SO}}{\text{NPO}}\times 100\%$$

### 2.5 Statistical analysis

Microsoft Excel 2010 was employed for statistical analysis and collation of the experimental data. One-way analysis of variance (ANOVA) was performed using the SPSS20.0 software.

## 3 Results

### 3.1 Identification of self-incompatibility in macadamia: field-bagging technology

#### 3.1.1 Identification of self-incompatibility in macadamia using field-bagging technology in 2021

To determine an effective method for identifying macadamia SI, we employed the field-bagging technique to assess eleven macadamia varieties in 2021 (**Supplementary Table 1**). The results of the comparison between the self-pollination initial nut set per raceme (SP_INS) and open-pollination initial nut set per raceme (OP_INS) (**Table 2**) indicate that the SP_INS were significantly lower than OP_INS in nine varieties (HY, A16, 816, 863, 820, D4, 842, D, and O.C.), with only two varieties (A4 and 246) showing similar values (no significant difference). The initial self-incompatibility index (I_SI) values, which were calculated from the SP_INS and OP_INS, indicate that the larger the I_SI, the lower the SP_INS, and the stronger the SI; conversely, the smaller the I_SI, the higher the SP_INS and the weaker the SI (**Table 2**). I_SI analysis showed that A16 and 816 were significantly stronger in SI than other varieties (P < 0.05), while A4 and 246 were significantly weaker (P < 0.05).

**Table 2:** Self-incompatibility in macadamia was evaluated in 2021 using the field-bagging method.

Variety	OP_INS	SP_INS	I_SI	OP_FNS	SP_FNS	F_SI	OP_AR	SP_AR
HY	0.83 ± 0.05	0.22 ± 0.04*	0.74 ± 0.04bcd	0.23 ± 0.03	0.03 ± 0.01*	0.86 ± 0.04bc	72.00 ± 3.94	84.62 ± 4.07
A16	0.25 ± 0.03	0.01 ± 0.01*	0.95 ± 0.03ab	0.14 ± 0.02	0 ± 0*	1.00 ± 0a	46.05 ± 7.33	100 ± 0*
A4	0.72 ± 0.11	0.87 ± 0.15	-0.21 ± 0.21f	0.15 ± 0.03	0.02 ± 0.01*	0.86 ± 0.04abc	79.53 ± 3.72	97.69 ± 0.67
816	1.47 ± 0.26	0.02 ± 0.01*	0.99 ± 0.01a	0.34 ± 0.05	0 ± 0*	0.99 ± 0.01ab	76.82 ± 3.22	80.00 ± 20
863	2.31 ± 0.34	0.77 ± 0.08*	0.66 ± 0.04cd	0.37 ± 0.05	0.13 ± 0.02*	0.64 ± 0.05defg	83.58 ± 2.4	82.76 ± 2.62
820	5.47 ± 0.7	2.54 ± 0.42*	0.54 ± 0.08d	0.13 ± 0.02	0.04 ± 0.01*	0.68 ± 0.08def	97.68 ± 0.37	98.43 ± 0.39
246	2.39 ± 0.43	2.04 ± 0.11	0.15 ± 0.04e	0.8 ± 0.07	0.37 ± 0.02*	0.53 ± 0.03g	66.62 ± 3.12	81.67 ± 1
D4	7.34 ± 0.26	3.41 ± 0.19*	0.54 ± 0.03d	0.34 ± 0.04	0.16 ± 0.02*	0.54 ± 0.05fg	95.32 ± 0.61	95.41 ± 0.54
842	4.16 ± 0.46	0.91 ± 0.06*	0.78 ± 0.01abc	0.57 ± 0.11	0.12 ± 0.02*	0.78 ± 0.04cd	86.38 ± 2.58	86.45 ± 2.56
D	3.52 ± 0.41	1.00 ± 0.14*	0.71 ± 0.04cd	0.66 ± 0.05	0.16 ± 0.03*	0.75 ± 0.05cde	81.23 ± 1.48	83.72 ± 3.47
O.C	4.44 ± 0.56	1.62 ± 0.22*	0.64 ± 0.05cd	0.28 ± 0.02	0.10 ± 0.02*	0.63 ± 0.07efg	93.76 ± 0.4	93.61 ± 1.25
Mean	2.99	1.22	0.59	0.36	0.10	0.75	79.91	89.49

OP, open-pollination; SP, self-pollination; INS, initial nut set per raceme; I_SI, index of the initial SI; FNS, final nut set per raceme; F_SI, index of final SI; AR, abscission rate; The significant difference between OP and SP was analyzed using the *t*-test, * indicates *p* ≤ 0.05. Data are presented as the mean ± standard deviation (n = 3). Significant differences were analyzed via ANOVA, different lowercase letters represent significant differences (P < 0.05). The same as **Tables 2**–**5**.

The final nut set per raceme (FNS) analysis indicated that the self-pollination final nut set per raceme (SP_FNS) values of all varieties were significantly lower than the open-pollination final nut set per raceme (OP_FNS) values, indicating that self-pollination significantly reduced the final nut setting rate. The final self-incompatibility index (F_SI) values, calculated from SP_FNS and OP_FNS, indicate that the larger the F_SI, the lower the SP_FNS and the stronger the SI; conversely, the smaller the F_SI, the higher the SP_FNS and the weaker the SI (**Table 2**). The F_SI analysis (**Table 2**) indicated that the SI of A16, 816, and A4 was strong, that the SI of HY, 842, D, 820, 863, and O.C. was intermediate, and that the SI of D4 and 246 was weak. Interestingly, the initial I_SI for A4 was the lowest, whereas its F_SI was exceptionally high. The analysis of abscission rates (AR) revealed high values under both open-pollination and self-pollination conditions.

#### 3.1.2 Identification of self-incompatibility in macadamia using field-bagging technology in 2022

To further validate the reliability of the field-bagging method for assessing SI in macadamia, we evaluated 26 macadamia varieties in 2022, including the 11 varieties previously studied in 2021 (**Table 3**). The results indicate that SP_INS were significantly lower than OP_INS in ten varieties (HY, 851, 918, 749, 932, 915, 842, D, 812, and O.C), suggesting that the initial nut set (INS) per raceme in these varieties was strongly influenced by self-pollination. In contrast, these were not significantly differences among the remaining varieties, indicating that the INS of these sixteen varieties were less affected by self-pollination. The I_SI indices of thirteen varieties (A16, A4, special, 904, 749, 836, 936, 863, 820, 246, 772, D4, and 814) were below the average of all tested varieties (0.41). In contrast, for thirteen varieties (HY, 695, 851, 918, 816, 932, 778, 915, 508, 842, D, 812, and O.C), this value was above the average (0.41). FNS analysis showed that the SP_FNS of nine varieties (A4, 695, 904, 749, 816, 932, 246, 772, and 842) were significantly less than their OP_FNS (P < 0.05); there were no significant differences among the other varieties. Varieties 816, 932, 812, 778, A4, 842, 904, and 820 either had an SP_FNS of zero or only had a few fruits, which indicates that their SI was strong. The SP_FNS of the varieties 863, 814, 915, and 936 was higher than their OP_FNS, and their F_SI was less than zero, indicating that their SI was weak. The SI of the other fourteen varieties (A16, special, 749, 836, 246, 772, D4, HY, 695, 851, 918, 508, D, and O.C) was at an intermediate level.

**Table 3:** Self-incompatibility analysis in macadamia in 2022 using the field-bagging method.

Variety	OP_INS	SP_INS	I_SI	OP_FNS	SP_FNS	F_SI	OP_AR	SP_AR
HY	28.00 ± 0.33	14.38 ± 0.82*	0.48 ± 0.03defgh	0.38 ± 0.07	0.10 ± 0.10	0.75 ± 0.25abc	98.66 ± 0.26	99.34 ± 0.66
A16	1.14 ± 0.14	0.92 ± 0.12	0.19 ± 0.1ijklm	0.32 ± 0.06	0.23 ± 0.12	0.27 ± 0.4cd	72.92 ± 5.21	75.48 ± 13.47
A4	33.54 ± 3.51	34.48 ± 6.73	-0.03 ± 0.2lm	0.75 ± 0.14	0.08 ± 0.08*	0.89 ± 0.11ab	97.76 ± 0.43	99.76 ± 0.24*
695	3.63 ± 0.99	1.07 ± 0.15	0.7 ± 0.04abcde	0.63 ± 0.13	0.34 ± 0.08*	0.45 ± 0.12abcd	82.76 ± 3.45	67.96 ± 7.25
Special	11.71 ± 1.14	8.11 ± 1.10	0.31 ± 0.09ghijk	0.83 ± 0.15	0.25 ± 0.07	0.69 ± 0.08abc	92.88 ± 1.28	96.84 ± 0.83
904	9.71 ± 3.16	10.01 ± 1.48	-0.05 ± 0.15lm	0.75 ± 0.14	0.13 ± 0.13*	0.83 ± 0.17abc	92.27 ± 1.49	98.77 ± 1.23*
851	24.38 ± 4.04	1.85 ± 0.33*	0.92 ± 0.01ab	0.33 ± 0.08	0.08 ± 0.08	0.75 ± 0.25abc	98.63 ± 0.34	95.56 ± 4.44
918	6.54 ± 0.72	3.77 ± 0.68*	0.44 ± 0.1efghi	0.29 ± 0.04	0.18 ± 0.05	0.39 ± 0.18bcd	95.54 ± 0.64	95.13 ± 1.46
749	12.88 ± 0.13	8.29 ± 1.29*	0.36 ± 0.1ghij	0.33 ± 0.08	0.09 ± 0.04*	0.73 ± 0.13abc	97.41 ± 0.65	98.92 ± 0.54
836	15.38 ± 2.25	9.27 ± 0.88	0.39 ± 0.06fghij	0.38 ± 0.07	0.23 ± 0.05	0.41 ± 0.13abcd	97.56 ± 0.47	97.64 ± 0.52
816	3.83 ± 2.22	0.10 ± 0.10	0.98 ± 0.02a	0.38 ± 0.07	0 ± 0*	1 ± 0a	90.22 ± 1.88	100 ± 0*
932	10.54 ± 2.24	0.29 ± 0.04*	0.97 ± 0a	0.42 ± 0.11	0 ± 0*	1 ± 0a	96.05 ± 1.05	100 ± 0*
936	8.56 ± 1.47	8.43 ± 0.74	0.02 ± 0.09lm	0.24 ± 0.01	0.26 ± 0.07	-0.06 ± 0.28d	97.19 ± 0.11	96.97 ± 0.80
863	3.08 ± 0.90	3.25 ± 0.42	-0.08 ± 0.14m	0.33 ± 0.08	0.98 ± 0.15	-1.96 ± 0.46f	89.19 ± 2.70	70.25 ± 4.66*
820	48.21 ± 2.86	41.58 ± 3.06	0.14 ± 0.06jklm	0.21 ± 0.04	0.04 ± 0.04	0.8 ± 0.2abc	99.57 ± 0.09	99.9 ± 0.10
778	6.71 ± 3.34	0.76 ± 0.14	0.89 ± 0.02ab	0.50 ± 0.13	0.04 ± 0.04	0.92 ± 0.08ab	92.55 ± 1.86	94.44 ± 5.56
915	30.5 ± 6.00	10.67 ± 1.80*	0.65 ± 0.06bcdef	0.29 ± 0.04	0.33 ± 0.04	-0.14 ± 0.14d	99.04 ± 0.14	96.88 ± 0.39*
508	18.38 ± 5.31	4.51 ± 0.79	0.76 ± 0.04abcd	0.50 ± 0.07	0.40 ± 0.15	0.21 ± 0.29d	97.28 ± 0.39	91.16 ± 3.27
246	5.54 ± 1.15	5.99 ± 1.26	-0.07 ± 0.23lm	0.67 ± 0.08	0.17 ± 0.04*	0.74 ± 0.06abc	87.97 ± 1.50	97.09 ± 0.66*
772	29.46 ± 2.69	23.02 ± 2.92	0.21 ± 0.1hijkl	0.71 ± 0.04	0.18 ± 0.05*	0.75 ± 0.08abc	97.6 ± 0.14	99.23 ± 0.23*
D4	19.79 ± 1.79	18.17 ± 3.76	0.08 ± 0.19klm	1.13 ± 0.19	0.97 ± 0.29	0.15 ± 0.26d	94.32 ± 0.96	94.72 ± 1.61
842	6.13 ± 0.52	1.67 ± 0.26*	0.73 ± 0.04abcd	0.58 ± 0.11	0.09 ± 0.04*	0.85 ± 0.08abc	90.48 ± 1.80	94.66 ± 2.69
D	12.54 ± 0.37	4.85 ± 0.05*	0.57 ± 0cdefg	0.79 ± 0.15	0.19 ± 0.19	0.76 ± 0.24abc	93.69 ± 1.20	96.44 ± 3.56
814	8.23 ± 1.74	12.99 ± 0.81	-0.58 ± 0.1n	1.92 ± 0.36	3.83 ± 0.58	-1.04 ± 0.31e	77.15 ± 4.39	70.49 ± 4.49
812	4.75 ± 0.71	0.76 ± 0.12*	0.84 ± 0.03abc	0.33 ± 0.08	0 ± 0	1 ± 0a	92.98 ± 1.75	100 ± 0*
O.C	10.42 ± 1.80	2.85 ± 0.47*	0.72 ± 0.05abcd	0.25 ± 0.07	0.08 ± 0.04	0.67 ± 0.17abc	97.6 ± 0.69	97.1 ± 1.45
Mean	14.37	8.92	0.41	0.55	0.36	0.45	93.05	93.26

The significant difference between OP and SP was analyzed by t-test, * indicates p ≤ 0.05. Data are presented as the mean ± standard deviation (n = 3). Significant differences were analyzed via ANOVA, different lowercase letters represent significant differences (P < 0.05).

As shown in **Table 3**, most varieties with high INS also exhibited high FNS. However, a few varieties (A4, 904, 820, and HY) displayed a higher INS but a lower FNS. Among these, variety A4 exhibited OP_INS and SP_INS of 33.54 and 34.48, respectively, while its OP_FNS and SP_FNS were as low as 0.75 and 0.08, respectively. Consequently, OP_AR and SP_AR were high, at 97.76% and 99.76%. AR analysis revealed that the OP_AR and SP_AR of nine varieties (A4, 904, 816, 932, 863, 915, 246, 772, and 812) reached a significant level; there were no significant differences in the other varieties (**Table 3**). In addition, among the 26 varieties included in the SI analysis, the SP_AR and OP-AR exceeded 90% in 21 and 22 varieties, respectively, indicating that both self-pollination and open-pollination resulted in a very high AR, which is consistent with the test results in 2021.

#### 3.1.3 Identification of self-incompatibility in macadamia using field-bagging technology in 2023

Macadamia pollination is significantly influenced by both biotic and abiotic stresses, such as bee population density, drought, high temperatures, diseases, and pests (Knight et al., 2005; McFadyen et al., 2011; Trueman, 2013; Grass et al., 2018; De Silva et al., 2024). Therefore, multi-year field experiments are essential to establish a reliable and accurate method for identifying SI. To further validate the reliability of the SI identification method in macadamia, fifteen representative varieties were selected for assessment in 2023. INS analysis revealed that the SP_INS of thirteen varieties (A16, special, 851, 816, 863, 820, 915, 246, D4, 842, D, 814, and O.C) were significantly lower than their OP_INS (**Table 4**), indicating that the INS of these varieties were strongly influenced by self-pollination. There were no significant differences between SP_INS and OP_INS in A4 and 828 (**Table 4**), indicating that the INS were less affected by self-pollination. The I_SI analysis showed that the 863, 814, D, 246, 851, and 816 varieties exhibited stronger SI, whereas the SI of A4 and 828 was weaker (**Table 4**). FNS analysis indicated that the OP_FNS were significantly higher than the SP_FNS in fourteen varieties (A16, A4, special, 851, 828, 816, 863, 820, 246, D4, 842, D, 814, and O.C) (P < 0.05); only in variety 915 was there no significant difference (**Table 4**). F_SI analysis showed that D4 and 851 had moderate SI levels, while 828 and 915 exhibited weak SI; the other varieties exhibited strong SI (A16, A4, special, 816, 863, 820, 246, 842, D, 814, and O.C). Interestingly, A4 exhibited a weak I_SI but a strong F_SI. AR analysis revealed that OP_AR was significantly lower than SP_AR in eight varieties (A4, special, 828, 816, 820, 842, D, and 814), whereas there was no significant difference between OP_AR and SP_AR in six varieties (A16, 851, 863, 915, 246, D4, and O.C) (**Table 4**). In addition, among the fifteen varieties for which SI was analyzed, the SP_AR and OP_AR exceeded 90% in eleven and thirteen varieties, respectively, indicating that both SP and OP were associated with a high AR, which is consistent with the test results in 2021 and 2022.

**Table 4:** Self-incompatibility analysis in macadamia in 2023 using the field-bagging method.

Variety	OP_INS	SP_INS	I_SI	OP_FNS	SP_FNS	F_SI	OP_AR	SP_AR
A16	4.36 ± 0.29	1.69 ± 0.15*	0.61 ± 0.03f	0.46 ± 0.03	0.11 ± 0.02*	0.76 ± 0.05bc	89.38 ± 0.77	93.43 ± 1.26
A4	12.55 ± 0.17	12.55 ± 0.98	0 ± 0.05i	0.51 ± 0.07	0.06 ± 0.01*	0.88 ± 0.02abc	95.91 ± 0.57	99.53 ± 0.09*
Special	9.79 ± 0.67	6.30 ± 0.12*	0.36 ± 0.01gh	0.52 ± 0.04	0 ± 0*	1 ± 0a	94.64 ± 0.40	100 ± 0*
851	12.24 ± 0.20	1.59 ± 0.22*	0.87 ± 0.02bc	0.21 ± 0.03	0.10 ± 0.01*	0.51 ± 0.06de	98.3 ± 0.21	93.55 ± 0.81
828	7.64 ± 0.11	7.52 ± 0.40	0.02 ± 0.05i	0.22 ± 0.02	0.13 ± 0.01*	0.41 ± 0.06e	97.07 ± 0.21	98.24 ± 0.17*
816	4.25 ± 0.16	0.89 ± 0.11*	0.79 ± 0.02cd	0.26 ± 0.02	0 ± 0*	1 ± 0a	93.85 ± 0.55	100 ± 0*
863	2.00 ± 0.06	0 ± 0*	1 ± 0a	0.22 ± 0.03	0 ± 0*	1 ± 0a	88.89 ± 1.25	NA
820	14.13 ± 0.14	9.93 ± 0.12*	0.3 ± 0.01h	0.16 ± 0.02	0 ± 0*	1 ± 0a	98.85 ± 0.11	100 ± 0*
915	10.5 ± 0.19	6.78 ± 0.16*	0.35 ± 0.02gh	0.2 ± 0.01	0.18 ± 0.03	0.11 ± 0.15f	98.09 ± 0.10	97.38 ± 0.43
246	2.44 ± 0.14	0.08 ± 0.01*	0.97 ± 0ab	0.06 ± 0.01	0.01 ± 0.01*	0.8 ± 0.2abc	97.58 ± 0.30	85.71 ± 14.29
D4	7.94 ± 0.11	4.42 ± 0.15*	0.44 ± 0.02g	0.17 ± 0.01	0.06 ± 0.01*	0.67 ± 0.07cd	97.89 ± 0.19	98.74 ± 0.25
842	7.12 ± 0.81	1.93 ± 0.33*	0.73 ± 0.05de	0.30 ± 0.04	0 ± 0*	1 ± 0a	95.82 ± 0.58	100 ± 0*
D	1.02 ± 0.09	0.03 ± 0.03*	0.97 ± 0.03a	0.19 ± 0.02	0.01 ± 0.01*	0.94 ± 0.06ab	81.52 ± 2.17	100 ± 0*
814	3.12 ± 0.17	0.03 ± 0.03*	0.99 ± 0.01a	0.44 ± 0.03	0 ± 0*	1 ± 0a	85.8 ± 0.85	100 ± 0*
O.C	2.51 ± 0.05	0.79 ± 0.10*	0.68 ± 0.04ef	0.12 ± 0.02	0.02 ± 0.01*	0.81 ± 0.09abc	95.34 ± 0.62	97.25 ± 1.38
Mean	6.77	3.64	0.6	0.27	0.05	0.79	93.93	97.42

The significant difference between OP and SP was analyzed by t-test, * indicates p ≤ 0.05. Data are presented as the mean ± standard deviation (n = 3). Significant differences were analyzed via ANOVA, different lowercase letters represent significant differences (P < 0.05).

#### 3.1.4 Identification of self-incompatibility in macadamia using field-bagging technology in 2024

Through a three-year (2021–2023) field-bagging experiment to assess SI in the macadamia germplasm, it was observed that both SP and OP exhibited extremely high AR and that the I_SI calculated from OP_INS and SP_INS were unreliable for evaluating SI in macadamia. In addition, measuring the INS is time-consuming and requires substantial manpower and material resources. Therefore, in 2024, we evaluated the SI of 23 macadamia varieties via field-bagging technology, recording only the FNS. As shown in **Table 5**, both OP_FNS and SP_FNS were low, with 11 varieties exhibiting an SP_FNS of zero. The difference analysis between SP_FNS and OP_FNS of the same species revealed no significant difference between HY and A4, whereas in the remaining varieties, SP_FNS was significantly lower than OP_FNS. F_SI analysis indicated that 19 varieties (A16, 695, special, 816, 936, 906, 862, 778, 951, 246, 772, D4, 842, D, 814, 812, O.C, 948, 861) exhibited strong SI, 2 varieties (508 and 828) showed moderate SI, and 2 varieties (A4 and HY) displayed weak SI.

**Table 5:** Self-incompatibility analysis in macadamia in 2024 using the field-bagging method.

Variety	OP_FNS	SP_FNS	F_SI
HY	0.12 ± 0.02	0.08 ± 0.02	0.29 ± 0.14e
A16	0.28 ± 0.05	0 ± 0*	1.00 ± 0a
A4	0.16 ± 0.02	0.11 ± 0.02	0.31 ± 0.14e
695	0.12 ± 0.02	0 ± 0*	1.00 ± 0a
Special	0.32 ± 0.02	0 ± 0*	1.00 ± 0a
828	0.16 ± 0.03	0.07 ± 0.01*	0.57 ± 0.06d
816	0.27 ± 0.02	0 ± 0*	1.00 ± 0a
936	0.36 ± 0.03	0.03 ± 0.01*	0.92 ± 0.02ab
906	0.13 ± 0.02	0.01 ± 0.01*	0.96 ± 0.04a
862	0.14 ± 0.01	0 ± 0*	1.00 ± 0a
778	0.38 ± 0.04	0.01 ± 0.01*	0.97 ± 0.03a
951	0.22 ± 0.01	0.02 ± 0.01*	0.93 ± 0.04ab
508	0.18 ± 0.01	0.05 ± 0.01*	0.73 ± 0.05c
246	0.11 ± 0.01	0 ± 0*	1.00 ± 0a
772	0.18 ± 0.02	0.04 ± 0.01*	0.79 ± 0.03bc
D4	0.22 ± 0.02	0 ± 0*	1.00 ± 0a
842	0.15 ± 0.02	0 ± 0*	1.00 ± 0a
D	0.09 ± 0.01	0.01 ± 0.01*	0.88 ± 0.06ab
948	0.06 ± 0.01	0.01 ± 0.01*	0.90 ± 0.1ab
861	0.16 ± 0.02	0 ± 0*	1.00 ± 0a
814	0.29 ± 0.02	0 ± 0*	1.00 ± 0a
812	0.18 ± 0.01	0.04 ± 0.01*	0.79 ± 0.03bc
O.C	0.25 ± 0.03	0 ± 0*	1.00 ± 0a
Mean	0.2	0.02	0.87

The significant difference between OP and SP was analyzed by t-test, * indicates p ≤ 0.05. Data are presented as the mean ± standard deviation (n = 3). Significant differences were analyzed via ANOVA, different lowercase letters represent significant differences (P < 0.05).

#### 3.1.5 Comparative analysis of the F_SI across different years

The results of a four-year (2021–2024) field-bagging experiment to assess SI in macadamia are presented in **Table 6**. Due to the high AR under both OP and SP, using I_SI to assess SI in macadamia is not reliable; therefore, F_SI were used to evaluate the degree of SI in macadamia. The results of the statistical analysis showed that the average value of OP_FNS was 0.37, the average value of SP_FNS was 0.17, and the average value of F_SI was 0.67 (**Table 6**). Therefore, based on the F_SI, we developed a standard for evaluating the SI of macadamia: F_SI ≥ 0.7 as strong SI, 0.4 ≤ F_SI < 0.7 as medium SI, and F_SI < 0.4 as weak SI. According to this evaluation standard, among the twenty-two varieties that were assessed in at least 2 years or more, thirteen varieties (816, 778, 842, Special, 812, D, 820, O.C, 246, 772, A16, A4 and 695) were identified as strong SI germplasms (F_SI ≥ 0.7), six varieties (HY, 851, D4, 828, 508, and 936) were identified as moderate SI germplasms (0.4 ≤ F_SI < 0.7), and 863, 915 and 814 were identified as weak SI germplasms (F_SI < 0.4) (**Table 6**). However, the F_SI of varieties 863 and 814 fluctuated significantly across years and even showed contradictory results; therefore, the degree of SI in these two varieties cannot be reliably determined by field-bagging technology.

**Table 6:** A four-year comparative analysis of F_SI in the field. M1 is the mean of one year. M2 is the mean of four years.

Variety	OP_FNS					SP_FNS					F_SI				
	2021	2022	2023	2024	Mean	2021	2022	2023	2024	Mean	2021	2022	2023	2024	Mean
HY	0.23	0.38	N	0.12	0.24	0.03	0.10	N	0.08	0.07	0.86	0.75	N	0.29	0.63
A16	0.14	0.32	0.46	0.28	0.30	0.00	0.23	0.11	0.00	0.08	1.00	0.27	0.76	1.00	0.76
A4	0.15	0.75	0.51	0.16	0.39	0.02	0.08	0.06	0.11	0.07	0.86	0.89	0.88	0.31	0.74
695	N	0.63	N	0.12	0.37	N	0.34	N	0.00	0.17	N	0.45	N	1.00	0.72
special	N	0.83	0.52	0.32	0.56	N	0.25	0.00	0.00	0.08	N	0.69	1.00	1.00	0.90
851	N	0.33	0.21	N	0.27	N	0.08	0.10	N	0.09	N	0.75	0.51	N	0.63
828	N	N	0.22	0.16	0.19	N	N	0.13	0.07	0.10	N	N	0.41	0.57	0.49
816	0.34	0.38	0.26	0.27	0.31	0.00	0.00	0.00	0.00	0.00	0.99	1.00	1.00	1.00	1.00
936	N	0.24	N	0.36	0.30	N	0.26	N	0.03	0.14	N	-0.06	N	0.92	0.43
863	0.37	0.33	0.22	N	0.31	0.13	0.98	0.00	N	0.37	0.64	-1.96	1.00	N	-0.11
820	0.13	0.21	0.16	N	0.17	0.04	0.04	0.00	N	0.03	0.68	0.80	1.00	N	0.83
778	N	0.50	N	0.38	0.44	N	0.04	N	0.01	0.03	N	0.92	N	0.97	0.94
915	N	0.29	0.20	N	0.25	N	0.33	0.18	N	0.26	N	-0.14	0.11	N	-0.02
508	N	0.50	N	0.18	0.34	N	0.40	N	0.05	0.22	N	0.21	N	0.73	0.47
246	0.80	0.67	0.06	0.11	0.41	0.37	0.17	0.01	0.00	0.14	0.53	0.74	0.80	1.00	0.77
772	N	0.71	N	0.18	0.45	N	0.18	N	0.04	0.11	N	0.75	N	0.79	0.77
D4	0.34	1.13	0.17	0.22	0.46	0.16	0.97	0.06	0.00	0.30	0.54	0.15	0.67	1.00	0.59
842	0.57	0.58	0.30	0.15	0.40	0.12	0.09	0.00	0.00	0.05	0.78	0.85	1.00	1.00	0.91
D	0.66	0.79	0.19	0.09	0.43	0.16	0.19	0.01	0.01	0.09	0.75	0.76	0.94	0.88	0.83
814	N	1.92	0.44	0.29	0.88	N	3.83	0.00	0.00	1.28	N	-1.04	1.00	1.00	0.32
812	N	0.33	N	0.18	0.26	N	0.00	N	0.04	0.02	N	1.00	N	0.79	0.89
O.C	0.28	0.25	0.12	0.25	0.22	0.10	0.08	0.02	0.00	0.05	0.63	0.67	0.81	1.00	0.78
M1	0.36	0.57	0.27	0.21	0.36	0.10	0.41	0.05	0.02	0.17	0.75	0.40	0.79	0.85	0.65
M2					0.37					0.17					0.67

### 3.2 Identification of self-incompatibility in macadamia by fluorescence-microscopy technology

To develop a method for identifying SI in macadamia that is more efficient, accurate, and cost-effective than the field-bagging method, we employed fluorescence-microscopy technology to assess 26 macadamia varieties that had previously been evaluated using the field-bagging technology. Among these varieties, the pistils of all individuals in clones 851, 816, 906, 820, 778, and D fell off 9 days after bagging, leaving no pistils for analysis and indicating that these clones have strong SI. The number of pistils remaining 9 days post-bagging for the remaining 20 varieties is presented in **Table 7**, with an average of 69 pistils for every nine racemes. Among the twenty varieties, in eight—842, A4, D4, 695, 863, 828, 849, and HY—the number of pistils was above the overall average, while the remaining varieties exhibited below-average pistil counts. Variety 842 had the highest pistil count at 161, followed by A4 with 106. In contrast, varieties 814, 812, and 246 all had fewer than 40 pistils.

**Table 7:** Analysis of pollen tube growth inside the style.

Variety	NPO (all)	NPT	US	MS	LS	SO	DO	NPO (mean)	PN/%	PUS/%	PMS/%	PLS/%	PSO/%	PDO/%
HY	77	0	7	22	48	51	26	26.00 ± 1.45	0.00 ± 0.00	10.14 ± 6.01	28.14 ± 3.04	61.71 ± 5.56a	66.06 ± 2.89	33.94 ± 2.89
A16	51	0	33	17	1	32	19	17.00 ± 1.15	0.00 ± 0.00	64.02 ± 12.49	33.76 ± 12.92	2.22 ± 2.22h	62.21 ± 6.09	37.79 ± 6.09
A4	106	17	34	47	8	84	22	35.00 ± 1.33	15.74 ± 3.97	32.61 ± 20.78	44.12 ± 17.23	7.53 ± 2.55gh	79.26 ± 1.71	20.74 ± 1.71
695	95	12	79	4	0	70	25	32.00 ± 1.20	12.79 ± 6.51	83.29 ± 5.55	3.92 ± 3.92	0.00 ± 0.00h	73.82 ± 1.76	26.18 ± 1.76
Special	60	12	35	10	3	39	21	20.00 ± 0.00	20.00 ± 5.77	58.33 ± 4.41	16.67 ± 4.41	5.00 ± 2.89gh	65.00 ± 5.77	35.00 ± 5.77
828	85	0	13	53	19	72	13	28.00 ± 0.88	0.00 ± 0.00	15.45 ± 6.69	62.63 ± 5.72	21.92 ± 7.37def	84.74 ± 0.72	15.26 ± 0.72
836	54	0	1	34	19	41	13	18.00 ± 1.15	0.00 ± 0.00	1.85 ± 1.85	62.27 ± 6.37	35.88 ± 5.62bc	76.62 ± 9.46	23.38 ± 9.46
936	57	11	13	15	18	42	15	19.00 ± 0.58	19.42 ± 6.38	22.57 ± 3.92	26.08 ± 4.73	31.93 ± 9.92cd	74.02 ± 7.69	25.98 ± 7.69
762	60	19	34	7	0	39	21	20.00 ± 0.00	31.67 ± 3.33	56.67 ± 6.01	11.67 ± 4.41	0.00 ± 0.00h	65.00 ± 7.64	35.00 ± 7.64
863	87	16	64	7	0	52	35	29.00 ± 2.08	17.70 ± 6.93	73.6 ± 4.88	8.70 ± 4.55	0.00 ± 0.00h	59.49 ± 2.08	40.51 ± 2.08
951	57	36	21	0	0	32	25	19.00 ± 0.58	62.81 ± 6.42	37.19 ± 6.42	0.00 ± 0.00	0.00 ± 0.00h	55.88 ± 4.62	44.12 ± 4.62
849	80	36	19	16	9	55	25	27.00 ± 0.88	45.06 ± 2.21	22.97 ± 11.82	20.7 ± 10.57	11.28 ± 0.38fgh	68.74 ± 0.81	31.26 ± 0.81
508	48	24	3	8	13	37	11	16.00 ± 1.15	52.05 ± 15.14	6.55 ± 3.62	15.81 ± 6.18	25.60 ± 12.21cde	76.88 ± 4.29	23.12 ± 4.29
246	39	23	7	6	3	26	13	13.00 ± 0.58	58.64 ± 7.30	18.25 ± 9.75	15.60 ± 11.68	7.51 ± 4.44gh	66.36 ± 7.14	33.64 ± 7.14
772	60	17	17	26	0	42	18	20.00 ± 0.58	28.63 ± 5.18	28.38 ± 1.90	42.99 ± 6.09	0.00 ± 0.00h	69.77 ± 6.43	30.23 ± 6.43
D4	97	0	22	65	10	70	27	32.00 ± 2.33	0.00 ± 0.00	24.44 ± 15.56	65.50 ± 14.91	10.06 ± 1.98fgh	71.47 ± 7.51	28.53 ± 7.51
842	161	72	61	27	1	104	57	54.00 ± 14.19	37.36 ± 15.04	34.75 ± 5.94	26.60 ± 19.44	1.28 ± 1.28h	64.10 ± 1.87	35.90 ± 1.87
814	23	0	2	14	7	11	12	8.00 ± 0.67	0.00 ± 0.00	8.47 ± 4.33	60.32 ± 8.84	31.22 ± 6.1cde	50.26 ± 24.94	49.74 ± 24.94
812	34	3	6	9	16	27	7	11.00 ± 0.33	8.84 ± 0.25	17.93 ± 5.47	26.26 ± 4.40	46.97 ± 1.52b	79.55 ± 5.72	20.45 ± 5.72
O.C	55	0	4	41	10	42	13	18.00 ± 1.45	0.00 ± 0.00	6.35 ± 6.35	75.50 ± 6.82	18.15 ± 0.75efg	76.59 ± 3.53	23.41 ± 3.53
Mean	69	15	24	21	9	48	21	23	20.54	31.01	32.36	15.91	69.29	30.71

NPO, the number of pistils utilized for observation; PT, pollen tube; PTL, length of the pollen tube; type I (NPT), the number of pistils with no PT inside the style; type II (US), the number of pistils where the pollen tube length is less than 2/10 of the style (arrest of pollen tube growth occurs in the upper part of the style), PTL ≤ 2/10; type III (MS), the number of pistils where the pollen tube length ranges from 2/10 to 7/10 of the style (arrest of pollen tube growth occurs in the mid-part of the style), 2/10 < PTL ≤ 7/10; type IV (LS), the number of pistils where the pollen tube length exceeds 7/10 of the style (arrest of pollen tube growth occurs in the lower part of the style), PTL > 7/10; SO, the number of ovaries with a single ovule; DO, the number of ovaries with double ovules; PN, percentage of NPT; PUS, percentage of US; PMS, percentage of MS; PLS, percentage of LS; PSO, percentage of ovaries with a single ovule; and PDO, percentage of ovaries with double ovules. Data are presented as the mean ± standard deviation. Significant differences were analyzed via ANOVA; different lowercase letters represent significant differences (p< 0.05).

**Figure 2:**
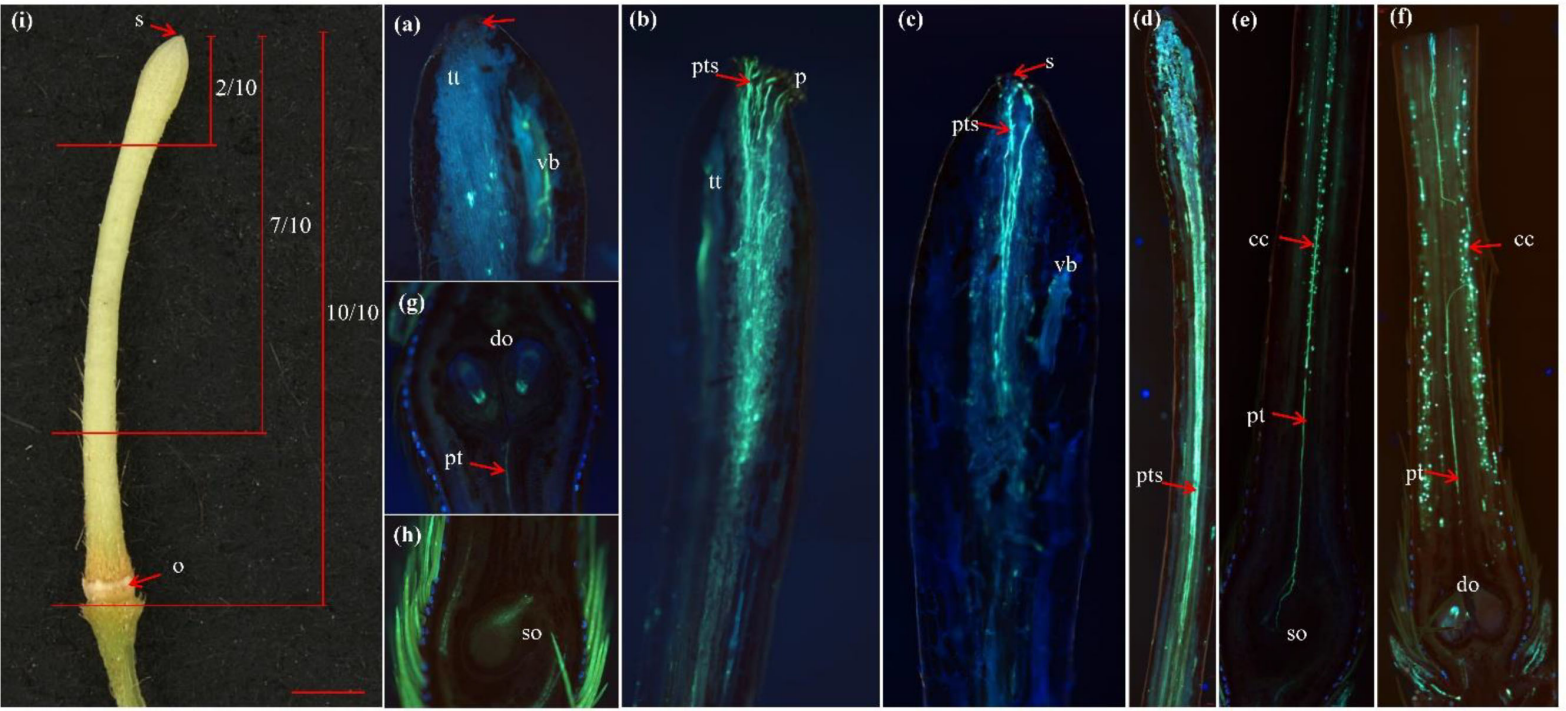
The growth of pollen tubes within the style. The growth of pollen tubes within the style. **(a)** no pollen tubes were observed in the stigma; **(b)** pollen tubes filled the entire stigma; **(c)** fewer pollen tubes were present in the stigma; **(d)** pollen tube growth arrested in the middle of the style; **(e)** pollen tubes successfully reached the base of the style; **(f)** pollen tubes entered the ovary; **(g)** single ovule (SO) ovary; **(h)** double ovaries (DO) ovary; **(i)** morphological characteristics and segmental divisions of the macadamia style. s, stigma; o, ovary; p, pollen; pt, single pollen tube; pts, several pollen tubes; vb, vascular bundle; tt, stylar transmitting tissue; cc, corpus callosum; so, single ovule; and do, double ovules.

Fluorescence microscopy revealed that the pollen tube growth within the style exhibited considerable variation among different varieties after 9 days of self-pollination. For instance, some varieties, such as 951, 246, 508, and 849 (**Table 7**; **Figure 2a**), had no pollen tubes in most pistil sections. In certain varieties, numerous pollen tubes were observed within the style, and nearly the entire stigma was filled with these pollen tubes in most pistil sections (**Figure 2b**). Some varieties exhibited fewer pollen tubes in the stigma in most pistil sections (**Figure 2c**), and in some varieties, pollen tubes were detected in the pistil style but growth had halted in the stigma or the upper part of the style in most pistil sections, for example, in 695, 863, A16, Special, and 762 (**Table 7**; **Figures 2b, c**). In some other varieties, pollen tubes were found in the pistil style and stagnated in the middle of the style in most of the pistil sections, such as O.C, D4, 828, 836, 814 (**Table 7**; **Figure 2d**). In varieties such as HY, 812, 836, 936, and 814 (**Table 7**; **Figures 2e, f**), most of the self-pollinated pollen tubes successfully reached the lower part of the style in most pistil sections, and in some cases, they extended into the ovary. In addition, we observed that most pollen tubes gradually ceased growth after entering the style, with only one or two pollen tubes reaching the base of the style (**Figures 2e, f**). After 9 days of self-pollination, in pistils where stigma had no pollen tubes and in pistils where the pollen tubes stagnated in the stigma, the upper or middle of the style typically abscised rapidly. Fertilization can only occur when pollen tubes successfully reach the base of the style or penetrated the ovary. Therefore, we evaluated SI by the percentage of pistils in which the pollen tube length exceeded seven-tenths of the style (PLS). A higher PLS value indicates that a greater proportion of pollen tubes have extended to the base of the style and entered the ovary, leading to an increased probability of self-fertilization, which in turn indicates weaker SI. Conversely, a lower PLS value corresponds to stronger SI.

As shown in **Table 7**, the average PLS value among the 20 varieties was 15.91%. Among these varieties, eight (HY, 828, 836, 936, 508, 814, 812, and O.C) exhibited PLS values higher than the average; the average PLS value for these varieties was 34.17%. Based on these findings, we established a fluorescence-microscopy-method standard for evaluating SI in macadamia using the PLS value: a PLS ≤ 16% indicates strong SI; a PLS between 16% and 35% (16% <PLS ≤ 35%) indicates medium SI; and a PLS > 35% indicates weak SI. According to this standard, 12 out of the 20 varieties were identified as strong SI; these varieties, ranked from strong to weak SI, were 772, 951, 863, 762, 695, 842, A16, Special, 246, A4, D4, and 849. Varieties 936, 814, 508, 828, and O.C were all identified as possessing a medium SI level and are sorted in descending order according to their intensity. Varieties HY, 836, and 812 were identified as weak SI.

Macadamia ovaries contain both a single ovule (SO) and double ovaries (DO) (**Figures 2g, h**). Single-seeded ovaries produce one round seed, whereas double ovaries yield two semi-circular seeds. The commercial value of semi-circular kernels is significantly lower than that of round kernels. Therefore, a higher proportion of single ovules within the ovaries is associated with greater commercial value. As shown in **Table 7**, the proportion of single-seeded ovules exceeds that of double-seeded ovules across all varieties.

## 4 Discussion

Macadamia is an important economic forest tree species (Stephenson and Cull, 1986; Trueman, 2013), known as the ‘king of nuts’. However, macadamia varieties exhibit either complete SI or partial self-incompatibility (PSI) (Sedgley et al., 2015; Trueman et al., 2022; De Silva et al., 2024), and also demonstrate cross-incompatibility (CI) (Herbert et al., 2019; Kong et al., 2023b), leading to low yields and poor kernel quality (Wallace et al., 1996; Richards et al., 2020; Kämper et al., 2021; Trueman et al., 2022). Previous studies have demonstrated that self-pollinated macadamia can set nuts, but the rate of this is extremely low (Wallace et al., 1996; Sedgley et al., 2015). Therefore, identifying the SI status of macadamia germplasms will highlight valuable parental resources for breeding new varieties with enhanced self-compatibility. However, to date, no reliable method has been established for accurately assessing the intensity of SI. To address this gap, the present study employed a field-bagging method to evaluate SI in macadamia germplasms. Furthermore, based on the results, a novel fluorescence-microscopy technology was developed to assess SI in macadamia.

Previous studies have conducted limited research on the self-pollination and hybridization rates of macadamia using the field-bagging method to isolate pollination (Trueman and Turnbull, 1994; Wallace et al., 1996; McFadyen et al., 2011; Sedgley et al., 2015); however, there are still no standardized methods or standards for identifying SI in macadamia. In this study, we identified the SI of macadamia germplasms via the field-bagging method of isolating pollination over four years. The results indicated that the open-pollination initial nut set per raceme (OP_INS) and the self-pollination initial nut set per raceme (SP_INS) values of all tested varieties were high from 2021 to 2023 (**Tables 2**-**4**), but the open-pollination final nut set per raceme (OP_FNS) and the self-pollination final nut set per raceme (SP_FNS) were low. Although there are more opportunities to accept heterologous pollen through open-pollination (OP) than through self-pollination (SP), the average abscission rates of open-pollination (OP_AR) and self-pollination (SP_AR) of all varieties over the four years were 90.43% and 93.57%, respectively. These results suggest that there is a high nut drop rate in both OP and SP. This phenomenon has also been widely reported (Trueman and Turnbull, 1994; McFadyen et al., 2011; Trueman, 2013; De Silva et al., 2024; Kaur et al., 2024; Trueman et al., 2024). However, its underlying mechanism remains unclear. Unfertilized small-fruit embryo abortion and tree nutritional limitations are two popular theories (Stephenson and Cull, 1986; Stephenson et al., 1989; Olesen et al., 2008; Herbert et al., 2019; Kämper et al., 2021; Trueman et al., 2022), but there is no conclusive evidence supporting either of the proposed mechanisms.

In this study, we employed the initial self-incompatibility index (I_SI), calculated from the OP_INS and SP_INS, to evaluate self-incompatibility (SI) in macadamia, and found its reliability to be low. We further assessed SI using the final self-incompatibility index (F_SI), derived from the OP_FNS and SP_FNS, which demonstrated higher reliability. Therefore, we established criteria for evaluating SI in macadamia based on the F_SI: F_SI ≥ 0.7 indicates strong SI, 0.4 ≤ F_SI < 0.7 indicates moderate SI, and F_SI < 0.4 indicates weak SI. According to these evaluation criteria, among the twenty-two varieties that were evaluated in at least two of the years, thirteen varieties with an average F_SI ≥ 0.7 were classified as strong-SI germplasms; namely, 816, 778, 842, Special, 812, D, 820, O.C, 246, 772, A16, A4, and 695. Six varieties (HY, 851, D4, 828, 508, and 936) exhibited moderate SI (0.4 ≤ F_SI < 0.7), while three varieties (863, 915, and 814) showed weak SI (F_SI < 0.4). However, it is particularly notable that the SI responses of varieties 863 and 814 varied significantly across years, displaying inconsistent phenotypic outcomes and even showing contradictory results. Therefore, the identification of SI via the field-bagging method failed for varieties 863 and 814. Previous studies have shown that 816, A16, and A4 have strong SI (Langdon et al., 2019; Richards et al., 2020; Kaur et al., 2024), and 508 has weak SI (Sedgley et al., 2015), which is consistent with the results of this study.

Comparative analysis of the OP_INS, SP_INS, OP_FNS, and SP_FNS across the same varieties in different years revealed that certain varieties exhibited high values of OP_INS and SP_INS. However, their OP_FNS and SP_FNS were consistently low, which is consistent with a previous study (Howell et al., 2018), and similar results have been reported in plum (Deng et al., 2022). That is, these varieties’ SI was weak according to I_SI, whereas their it was strong according to F_SI; this trend was particularly prominent in varieties A4 and 820. From 2021 to 2023, the average values of I_SI, F_SI, OP_AR, and SP_AR in variety A4 were −0.08, 0.87, 91.07%, and 98.99%, respectively. These results indicate that the SI of A4 is extremely strong. Currently, the underlying mechanism causing the phenomenon of a very high initial nut set but a very low final nut set remains unclear (Kaur et al., 2024, Kaur et al., 2026). We speculate that this phenomenon may result from substances that are produced during the accumulation of self-pollen germinating on the stigma, its growth into the style, and from pollen tube growth and elongation, which leads to a delay in pistil shedding.

The field-bagging method for studying SI in macadamia involves preventing cross-pollination and promoting self-pollination, with the nut set rate under self-pollination used as the key indicator to evaluate SI. However, the final nut set rate is influenced by external environmental conditions, tree nutritional status, and pest and disease pressures, resulting in significant inter annual variation. Therefore, multiple years of experimental data are required to reliably determine a variety’s SI phenotype. To develop a faster, more accurate and more economical method to identify the SI of macadamia than the field-bagging method, we developed a fluorescence-microscopy method, assessing the SI in 26 macadamia varieties. Sedgley et al (Sedgley, 1983; Sedgley et al., 1985) reported that pollen tubes could reach the ovary 7–9 days after flowering, and our previous findings further corroborated this observation (Kong et al., 2023a). Therefore, in this study, we examined pistils at 9 days post-pollination to assess SI across different macadamia varieties. Based on the percentage of pistils in which the pollen tube length exceeds 7/10 of the style (PLS), we established standardized criteria for evaluating SI in macadamia: PLS ≤ 16% indicates strong SI; 16% < PLS ≤ 35% indicates moderate SI; and PLS > 35% indicates weak SI. Among the twenty varieties, twelve were identified as having strong SI (PLS ≤ 16%), ranked from strongest to weakest as follows: 772, 951, 863, 762, 695, 842, A16, Special, 246, A4, D4, and 849. Additionally, all pistils of six varieties (851, 816, 906, 820, 778, and D) evaluated using fluorescence microscopy fell off after 9 days of raceme bagging, and they were directly determined to have strong SI. Ultimately, 18 varieties were identified as having strong SI by fluorescence microscopy. Varieties 936, 814, 508, 828, and O.C were classified as having moderate SI; HY, 836, and 812 were identified as having low SI. The results of SI intensity assessments in macadamia varieties using fluorescence microscopy were consistent with previous research results (Sedgley et al., 2015; Langdon et al., 2019; Richards et al., 2020; Kaur et al., 2024). The characterization of self-incompatibility in macadamia germplasm is pivotal for targeted breeding. By leveraging this trait, breeders can select parental lines with lower self-incompatibility for controlled hybridization, thereby enhancing the likelihood of generating novel cultivars with strong self-compatibility.

The number of remaining pistils in the 26 macadamia varieties observed under fluorescence microscopy was significantly different after 9 days of self-pollination (**Table 7**). Variety 842 retained the highest number of pistils, at 161, followed by A4, which had 106 pistils; in contrast, the number of pistils in 814, 812, and 246 was below 40. Additionally, no pistils were collected for fluorescence-microscopy observation from six varieties (851, 816, 906, 820, 778, D). Based on the number of pistils retained in the raceme, we initially speculated that the varieties with more retained pistils (842, A4) may have weaker SI, and the varieties with lower pistil retention (814, 812, and 246) may have stronger SI. However, the results of fluorescence-microscopy observations were contrary to our speculation: the SI of 842 and A4 were stronger and the SI of 814, 812 and 246 was weaker. Field observations also showed that the SI of 842 and A4 were stronger than that of 814 and 246. Why does the variety with more remaining pistils have stronger SI 9 days after flowering? What causes their pistils to remain longer on the raceme axis? These issues deserve ongoing attention.

A comparison of the fluorescence-microscopy and field-bagging methods for identifying SI in macadamia revealed that the cost of the latter for evaluating SI in a single macadamia variety is CNY 250 per year. Moreover, 2–4 years of evaluation data are required to assess the SI of a variety for field bagging, resulting in a total cost of CNY 500–1,000 per variety. Fluorescence microscopy only requires approximately CNY 35 per year to determine the SI level for a single macadamia variety, with preliminary SI assessment achievable within one growing season. The fluorescence-microscopy method has several advantages over the field-bagging method. The cost of the field-bagging method is 14 to 29 times higher than that of fluorescence microscopy. Fluorescence microscopy enables intuitive, rapid, and precise evaluation of SI intensity across macadamia varieties, and it requires minimal labor, produces reliable results, and has substantially reduced time and economic costs. Therefore, fluorescence microscopy is a novel approach and offers essential technical support for assessing SI levels in macadamia germplasms. Additionally, this method provides a valuable reference for the identification of the self-(in)compatibility in other crop species.

## 5 Conclusions

Over a four-year period, we evaluated SI in macadamia using the field-bagging method, establishing the SI intensity according to the F_SI index: F_SI ≥ 0.7 indicates strong SI, 0.4 ≤ F_SI < 0.7 indicates moderate SI, and F_SI < 0.4 indicates weak SI. Additionally, based on the fluorescence-microscopy method, we established a classification system for macadamia SI levels using PLS values: PLS ≤ 16% indicates strong SI; 16% < PLS ≤ 35% indicates moderate SI; PLS > 35% indicates weak SI. Through comprehensive analysis of the results of field-bagging and fluorescence-microscopy observations, thirteen strong SI varieties (816, 778, 842, Special, 812, D, 820, 246, 772, A16, A4, 951 and 695), six moderate SI varieties (851, 828, 508, 936, O.C, and D4), and four weak-SI varieties (915, HY, 836, and 814) were identified. In this study, the large-scale SI identification and classification of macadamia germplasm resources were systematically carried out for the first time, which filled the gap of basic data in this field. Additionally, we established an efficient, standardized SI identification technology system, which solved the problem of low efficiency of traditional methods. Our results provide a solid theoretical basis and technical support for the directional breeding of self-compatible varieties of macadamia.

## Data Availability

The original contributions presented in the study are included in the article/**Supplementary Material**. Further inquiries can be directed to the corresponding author.
