# Supplementary material for: Identification of self-incompatibility in macadamia (Macadamia SPP.) using field-bagging and fluorescence microscopy
Source: Front Plant Sci. 2026 Apr 16;17:1771293. doi: 10.3389/fpls.2026.1771293 (PMC13128622; doi:10.3389/fpls.2026.1771293)
Supplement: Supplementary file 1 [file Table1.docx]

**Table S1.** Detailed information of the varieties and pollination in 2021–2024. SP: self-pollination; OP: open-pollination, and rep: replicate.

| Year | Number of varieties | Number of SP (bagged) racemes | | |  | Number of OP racemes | | |
| --- | --- | --- | --- | --- | --- | --- | --- | --- |
|  |  | rep 1 | rep 2 | rep 3 |  | rep 1 | rep 2 | rep 3 |
| 2021 | 11 | 100 | 100 | 100 |  | 100 | 100 | 100 |
| 2022 | 26 | 8 | 8 | 8 |  | 8 | 8 | 8 |
| 2023 | 15 | 30 | 30 | 30 |  | 30 | 30 | 30 |
| 2024 | 23 | 60 | 60 | 60 |  | 60 | 60 | 60 |
